# (Al_13.28_Si_2.72_)(Fe_1.19_Ni_2.81_)

**DOI:** 10.1107/S2414314625010387

**Published:** 2025-11-21

**Authors:** Mei Chen, Changzeng Fan, Bin Wen, Lifeng Zhang

**Affiliations:** a State Key Laboratory of Metastable Materials Science and Technology Yanshan University,Qinhuangdao 066004 People’s Republic of China; b Hebei Key Lab for Optimizing Metal Product Technology and Performance Yanshan University,Qinhuangdao 066004 People’s Republic of China; c School of Mechanical and Materials Engineering North China University of Technology,Beijing 100144 People’s Republic of China; Vienna University of Technology, Austria

**Keywords:** crystal structure, high-pressure, inter­metallic, Al_13.28_Fe_1.19_Ni_2.81_Si_2.72_ phase

## Abstract

The inter­metallic phase (Al_13.28_Si_2.72_)(Fe_1.19_Ni_2.81_) was obtained by high-pressure sinter­ing (HPS) of a mixture with an elemental atomic ratio corresponding to (Al,Si)_5_(Fe,Ni). The space group was determined to be *C*2/*m* with *Z* = 2. The structure model contains three co-occupied sites with occupancy ratios of Si:Al = 0.679:0.321, Ni:Fe = 0.46:0.54 and Ni:Fe = 0.94:0.06, and five sites with full occupancy of Al atoms.

## Abstract 2

The crystal structure of (Al_13.28_Si_2.72_)(Fe_1.19_Ni_2.81_) comprises eight sites in the asymmetric unit, one of which is co-occupied by pairs of (Al,Si) atoms, and two by pairs of (Fe,Ni) atoms.

## Graphical abstract

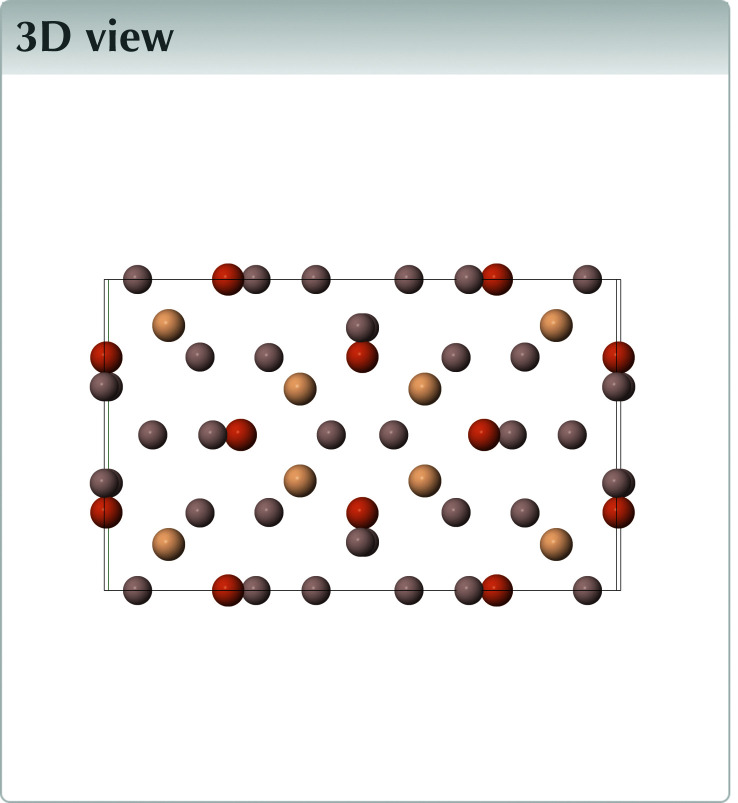

## Structure description

In the quaternary Al–Si–Ni–Fe near-eutectic alloy, an (Al,Si)_5_(Fe,Ni) phase exists, which adopts the tetra­gonal (Al_2.7_Si_2.3_)Fe structure type. The reported composition is based on TEM-EDX point analysis with atomic percentages of 77.1 (Al), 6.5 (Si), 8.6 (Fe) and 7.8 (Ni) (Cai *et al.*, 2023 ▸). It has been confirmed through first-principles calculations that the Si sites tend to be co-occupied by Al atoms and are adjacent to the Fe atoms; the co-occupied atoms have stable configurations (Cai *et al.*, 2023 ▸). The growth mechanism of this inter­metallic phase under high-pressure and high-temperature (HPHT) conditions was investigated by the high-pressure sinter­ing (HPS) process, which eventually led to a new phase with composition (Al_13.28_Si_2.72_)(Fe_1.19_Ni_2.81_) in the quaternary Al–Si–Ni–Fe system. The title phase and the Al_13_Fe_4_ phase (Grin *et al.*, 1994 ▸) both crystallize in space group type *C*2/*m*, however without close relationship. The deca­gonal quasicrystal approximant Al_76_Ni_9_Fe_15_, studied by Nejadsattari *et al.* (2016 ▸) in the Al–Ni–Fe ternary system, is isotypic with Al_13_Fe_4_, where Fe and Ni atoms co-occupy 4*i* and 8*j* sites.

**Figure 1:**
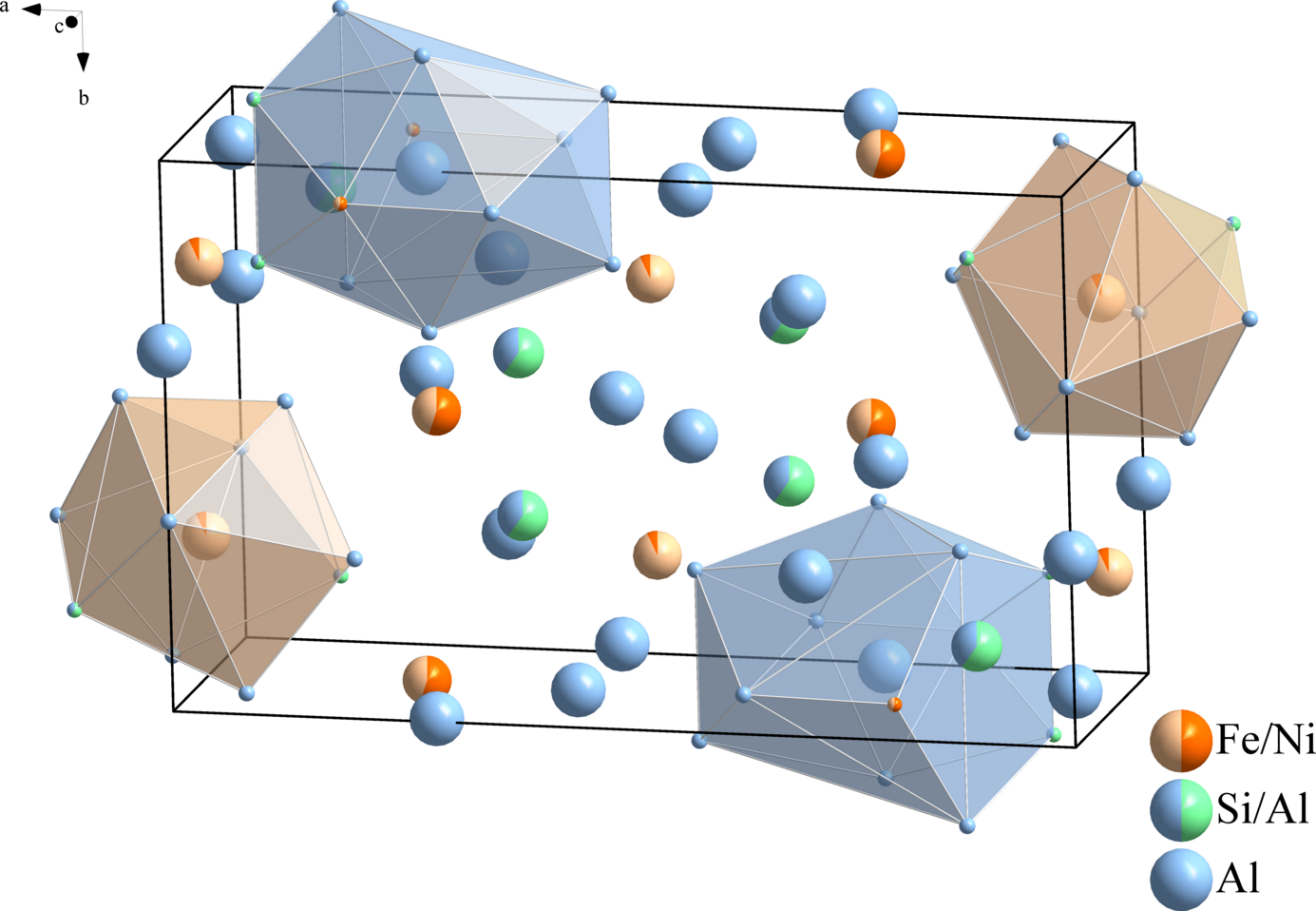
The crystal structure of (Al_13.28_Si_2.72_)(Fe_1.19_Ni_2.81_) with two Al4 atoms on the 4*i* site and two (Fe,Ni)2 atoms on the 4*h* site displayed with their coordination environments as polyhedra.

The asymmetric unit of (Al_13.28_Si_2.72_)(Fe_1.19_Ni_2.81_) comprises eight sites: five are fully occupied by Al atoms at Wyckoff sites 8*j* (Al2), 4*g* (Al3), and 4*i* (Al4, Al5, Al6); one site (8*j*) is partially occupied by Si1 (occupancy 0.679) and Al1 (occupancy 0.321) atoms; one site (4*i)* is partially occupied by Ni1 (occupancy 0.46) and Fe1 (occupancy 0.54) atoms; one site (4*h*) is partially occupied by Ni2 (occupancy 0.94) and Fe2 (occupancy 0.06). The Al4 atom at the 4*i* site and the (Fe,Ni)2 atoms at the co-occupied 4*h* site are surrounded by twelve and ten atoms, forming distorted 19-face and 16-face polyhedra, respectively (Figs. 1 ▸–3 ▸ ▸).

## Synthesis and crystallization

The high purity elements aluminium (indicated purity 99.9%; 0.6739 g), iron (indicated purity 99.9%; 0.1442 g), nickel (indicated purity 99.9%; 0.1566 g), and silicon (indicated purity 99.9%; 0.0826 g) were evenly mixed with a stoichiometric ratio of 77.1: 8.6: 7.8: 6.5 and ground in an agate mortar for 40 min. The mixed powder was then placed in a cemented carbide grinding mold with a diameter of 5 mm and pressed into a block at about 4 MPa for three min. Cylindrical blocks without deformation and cracks were obtained. Details of high-pressure sinter­ing experiments using six-anvil high-temperature and high-pressure equipment are described in detail (Liu & Fan, 2018 ▸). The sample was pressurized to 6 GPa and heated at 1676 K for 30 min., then cooled to 1131 K and held for 60 min., and finally rapidly cooled to room temperature by turning off the furnace power. A single-crystal was selected and mounted on a glass fiber for SXRD measurement.

## Refinement

Crystal data, data collection and structure refinement details are summarized in Table 1 ▸. Due to the similar electron densities of Ni/Fe and Si/Al at co-occupied sites, the site-occupancy refinement for these mixed sites is inherently inaccurate. To obtain a reasonable composition, the total proportion of Fe and Ni was maintained at approximately 20% (to preserve structural validity), while the relative occupancies of Al and Si were adjusted to approximate their ratio determined from EDX data (see the supporting information) as closely as possible. Several compositions were evaluated, and the model with the composition Al 66.42%, Si 13.58%, Fe 5.97%, Ni 14.03% was finally selected. This model maintains structural rationality, closely approximates the composition determined by EDX, and yields satisfactory reliability factors. The maximum and minimum residual electron densities in the final difference map are located 1.50 Å from (Al,Si)1 and 0.93 Å from Al4, respectively.

## Supplementary Material

Crystal structure: contains datablock(s) I. DOI: 10.1107/S2414314625010387/wm4239sup1.cif

Structure factors: contains datablock(s) I. DOI: 10.1107/S2414314625010387/wm4239Isup2.hkl

supplementary marerials. DOI: 10.1107/S2414314625010387/wm4239sup3.docx

CCDC reference: 2503943

Additional supporting information:  crystallographic information; 3D view; checkCIF report

## Figures and Tables

**Figure 2:**
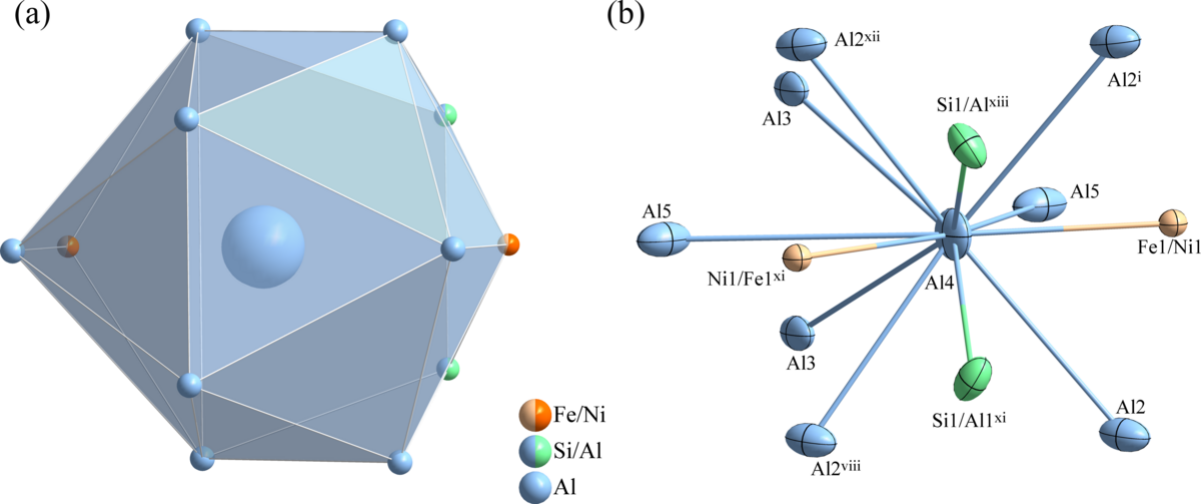
(*a*) The enneadeca­hedron formed around the Al4 atom at the 4*i* site; (*b*) the environment of the Al4 atom with displacement ellipsoids given at the 80% probability level. [Symmetry codes: (i) *x*, −*y* + 1, *z*; (viii) −*x* + 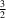, −*y* + 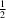, −*z*; (xi) *x*, *y*, *z* − 1; (xii) −*x* + 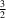, *y* + 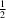, −*z*; (xiii) *x*, −*y* + 1, *z* − 1.]

**Figure 3:**
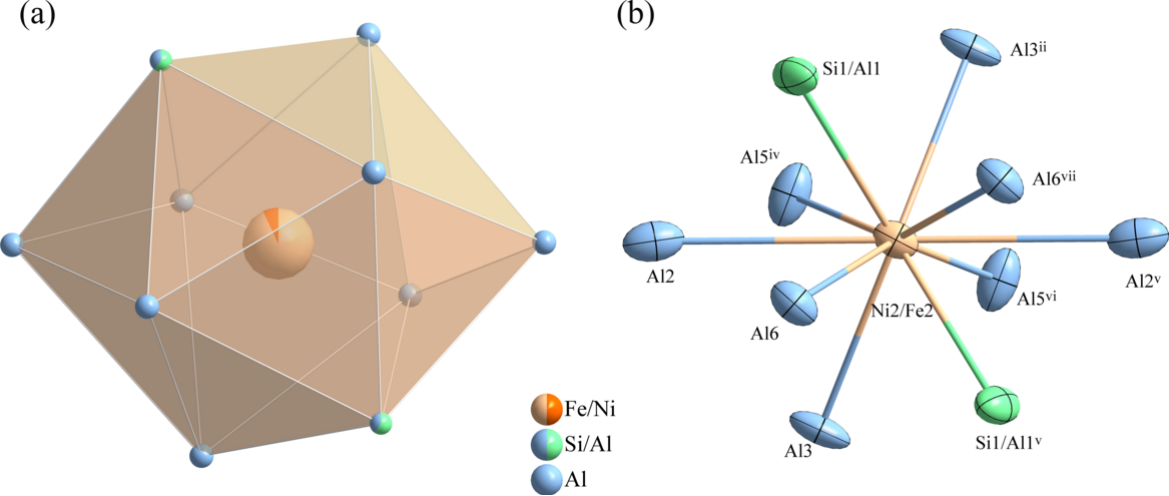
(*a*) The hexa­deca­hedron formed around the (Fe,Ni)2 atoms at the 4*h* site; (*b*) the environment of the (Fe,Ni)2 atoms with displacement ellipsoids given at the 90% probability level. [Symmetry codes: (ii) *x*, *y*, *z* + 1; (iv) −*x* + 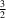, −*y* + 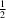, −*z* + 1; (v) −*x* + 1, *y*, −*z* + 1; (vi) *x* − 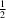, *y* − 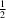, *z*; (vii) −*x* + 1, −*y* + 1, −*z* + 1.]

**Table 1:** Experimental details

Crystal data	
Chemical formula	Al_13.28_Fe_1.19_Ni_2.81_Si_2.72_
*M*_r_	666.12
Crystal system, space group	Monoclinic, *C*2/*m*
Temperature (K)	296
*a*, *b*, *c* (Å)	14.2407 (16), 8.6413 (10), 4.7167 (5)
β (°)	91.359 (4)
*V* (Å^3^)	580.27 (11)
*Z*	2
Radiation type	Mo *K*α
μ (mm^−1^)	7.20
Crystal size (mm)	0.10 × 0.09 × 0.08

Data collection	
Diffractometer	Bruker D8 Venture Photon 100 CMOS
Absorption correction	Multi-scan (*SADABS*; Krause *et al.*, 2015 ▸)
*T*_min_, *T*_max_	0.600, 0.746
No. of measured, independent and observed [*I* > 2σ(*I*)] reflections	3712, 707, 586
*R*_int_	0.057
(sin θ/λ)_max_ (Å^−1^)	0.650

Refinement	
*R*[*F*^2^ > 2σ(*F*^2^)], *wR*(*F*^2^), *S*	0.036, 0.059, 1.11
No. of reflections	707
No. of parameters	55
Δρ_max_, Δρ_min_ (e Å^−3^)	0.81, −0.83

Computer programs: *APEX5* and *SAINT* (Bruker, 2023 ▸), *SHELXT* (Sheldrick, 2015*a* ▸), *SHELXT* (Sheldrick, 2015*b* ▸), *DIAMOND* (Brandenburg & Putz, 2017 ▸) and *publCIF* (Westrip, 2010 ▸).

## full crystallographic data

### Aluminium iron nickel silicide Crystal data

**Table:**

Al_13.28_Fe_1.19_Ni_2.81_Si_2.72_	*F*(000) = 641
*M**_r_* = 666.12	*D*_x_ = 3.812 Mg m^−^^3^
Monoclinic, *C*2/*m*	Mo *K*α radiation, λ = 0.71073 Å
*a* = 14.2407 (16) Å	Cell parameters from 2307 reflections
*b* = 8.6413 (10) Å	θ = 2.8–27.5°
*c* = 4.7167 (5) Å	µ = 7.20 mm^−^^1^
β = 91.359 (4)°	*T* = 296 K
*V* = 580.27 (11) Å^3^	Lump, gray
*Z* = 2	0.10 × 0.09 × 0.08 mm

### Aluminium iron nickel silicide Data collection

**Table:**

Bruker D8 Venture Photon 100 CMOS diffractometer	586 reflections with *I* > 2σ(*I*)
phi and ω scans	*R*_int_ = 0.057
Absorption correction: multi-scan (SADABS; Krause et al., 2015)	θ_max_ = 27.5°, θ_min_ = 2.8°
*T*_min_ = 0.600, *T*_max_ = 0.746	*h* = −18→18
3712 measured reflections	*k* = −10→11
707 independent reflections	*l* = −5→6

### Aluminium iron nickel silicide Refinement

**Table:**

Refinement on *F*^2^	55 parameters
Least-squares matrix: full	0 restraints
*R*[*F*^2^ > 2σ(*F*^2^)] = 0.036	*w* = 1/[σ^2^(*F*_o_^2^) + (0.0135*P*)^2^ + 2.4081*P*] where *P* = (*F*_o_^2^ + 2*F*_c_^2^)/3
*wR*(*F*^2^) = 0.059	(Δ/σ)_max_ < 0.001
*S* = 1.11	Δρ_max_ = 0.81 e Å^−^^3^
707 reflections	Δρ_min_ = −0.83 e Å^−^^3^

### Aluminium iron nickel silicide Special details

**Table:**

Geometry. All esds (except the esd in the dihedral angle between two l.s. planes) are estimated using the full covariance matrix. The cell esds are taken into account individually in the estimation of esds in distances, angles and torsion angles; correlations between esds in cell parameters are only used when they are defined by crystal symmetry. An approximate (isotropic) treatment of cell esds is used for estimating esds involving l.s. planes.

### Aluminium iron nickel silicide Fractional atomic coordinates and isotropic or equivalent isotropic displacement parameters (Å^2^)

**Table:**

	*x*	*y*	*z*	*U*_iso_*/*U*_eq_	Occ. (<1)
Ni1	0.73802 (5)	0.500000	0.53636 (18)	0.0060 (3)	0.46 (5)
Fe1	0.73802 (5)	0.500000	0.53636 (18)	0.0060 (3)	0.54 (5)
Ni2	0.500000	0.24889 (9)	0.500000	0.0076 (3)	0.94 (5)
Fe2	0.500000	0.24889 (9)	0.500000	0.0076 (3)	0.06 (5)
Si1	0.62407 (8)	0.35232 (15)	0.7917 (3)	0.0110 (3)	0.679
Al1	0.62407 (8)	0.35232 (15)	0.7917 (3)	0.0110 (3)	0.321
Al2	0.68103 (9)	0.25081 (16)	0.2938 (3)	0.0116 (3)	
Al3	0.500000	0.1550 (2)	0.000000	0.0111 (5)	
Al4	0.78856 (14)	0.500000	0.0425 (4)	0.0138 (5)	
Al5	0.90936 (13)	0.500000	0.4897 (4)	0.0127 (5)	
Al6	0.55911 (13)	0.500000	0.2742 (4)	0.0097 (4)	

### Aluminium iron nickel silicide Atomic displacement parameters (Å^2^)

**Table:**

	*U*^11^	*U*^22^	*U*^33^	*U*^12^	*U*^13^	*U*^23^
Ni1	0.0077 (5)	0.0047 (4)	0.0055 (5)	0.000	0.0007 (3)	0.000
Fe1	0.0077 (5)	0.0047 (4)	0.0055 (5)	0.000	0.0007 (3)	0.000
Ni2	0.0113 (4)	0.0052 (4)	0.0065 (5)	0.000	0.0008 (3)	0.000
Fe2	0.0113 (4)	0.0052 (4)	0.0065 (5)	0.000	0.0008 (3)	0.000
Si1	0.0115 (7)	0.0135 (7)	0.0081 (7)	−0.0040 (5)	−0.0005 (5)	0.0027 (6)
Al1	0.0115 (7)	0.0135 (7)	0.0081 (7)	−0.0040 (5)	−0.0005 (5)	0.0027 (6)
Al2	0.0168 (7)	0.0071 (6)	0.0107 (7)	0.0012 (6)	−0.0046 (5)	−0.0007 (6)
Al3	0.0218 (11)	0.0074 (10)	0.0042 (10)	0.000	0.0015 (8)	0.000
Al4	0.0198 (10)	0.0172 (11)	0.0044 (11)	0.000	−0.0002 (8)	0.000
Al5	0.0080 (9)	0.0075 (10)	0.0225 (12)	0.000	0.0004 (8)	0.000
Al6	0.0138 (9)	0.0075 (10)	0.0079 (10)	0.000	0.0030 (8)	0.000

### Aluminium iron nickel silicide Geometric parameters (Å, º)

**Table:**

Ni1—Si1^i^	2.4093 (13)	Ni2—Al2^v^	2.7768 (13)
Ni1—Si1	2.4094 (13)	Fe2—Si1	2.3872 (13)
Ni1—Al4	2.455 (2)	Fe2—Si1^v^	2.3872 (13)
Ni1—Al5	2.455 (2)	Fe2—Al3^ii^	2.4939 (7)
Ni1—Al4^ii^	2.477 (2)	Fe2—Al3	2.4939 (7)
Ni1—Al2^i^	2.5612 (14)	Fe2—Al5^vi^	2.5082 (12)
Ni1—Al2	2.5612 (14)	Fe2—Al5^iv^	2.5082 (12)
Ni1—Al2^iii^	2.5737 (14)	Fe2—Al6	2.5679 (12)
Ni1—Al2^iv^	2.5737 (14)	Fe2—Al6^vii^	2.5680 (12)
Ni1—Al6	2.806 (2)	Fe2—Al2	2.7768 (13)
Fe1—Si1^i^	2.4093 (13)	Fe2—Al2^v^	2.7768 (13)
Fe1—Si1	2.4094 (13)	Si1—Si1^i^	2.552 (3)
Fe1—Al4	2.455 (2)	Si1—Al2^ii^	2.6354 (19)
Fe1—Al5	2.455 (2)	Si1—Al2	2.6518 (19)
Fe1—Al4^ii^	2.477 (2)	Si1—Al3^ii^	2.6601 (17)
Fe1—Al2^i^	2.5612 (14)	Si1—Al6^ii^	2.786 (2)
Fe1—Al2	2.5612 (14)	Si1—Al6	2.887 (2)
Fe1—Al2^iii^	2.5737 (14)	Si1—Al4^ii^	2.895 (2)
Fe1—Al2^iv^	2.5737 (14)	Si1—Al6^vii^	2.914 (2)
Fe1—Al6	2.806 (2)	Al2—Al4^viii^	2.7264 (18)
Ni2—Si1	2.3872 (13)	Al2—Al5^iv^	2.7311 (18)
Ni2—Si1^v^	2.3872 (13)	Al2—Al2^iv^	2.731 (3)
Ni2—Al3^ii^	2.4939 (7)	Al2—Al6	2.7663 (18)
Ni2—Al3	2.4939 (7)	Al3—Al3^ix^	2.679 (4)
Ni2—Al5^vi^	2.5082 (12)	Al3—Al5^viii^	2.990 (2)
Ni2—Al5^iv^	2.5082 (12)	Al3—Al5^vi^	2.990 (2)
Ni2—Al6	2.5679 (12)	Al4—Al5	2.690 (3)
Ni2—Al6^vii^	2.5680 (12)	Al5—Al5^x^	2.581 (4)
Ni2—Al2	2.7768 (13)	Al6—Al6^vii^	2.747 (4)
			
Si1^i^—Ni1—Si1	63.97 (7)	Al2—Si1—Al6	59.74 (5)
Si1^i^—Ni1—Al4	133.68 (5)	Al3^ii^—Si1—Al6	113.15 (5)
Si1—Ni1—Al4	133.68 (5)	Al6^ii^—Si1—Al6	112.47 (6)
Si1^i^—Ni1—Al5	136.66 (5)	Ni2—Si1—Al4^ii^	168.83 (6)
Si1—Ni1—Al5	136.66 (5)	Ni1—Si1—Al4^ii^	54.75 (5)
Al4—Ni1—Al5	66.45 (7)	Si1^i^—Si1—Al4^ii^	63.85 (3)
Si1^i^—Ni1—Al4^ii^	72.66 (5)	Al2^ii^—Si1—Al4^ii^	63.31 (5)
Si1—Ni1—Al4^ii^	72.66 (5)	Al2—Si1—Al4^ii^	104.24 (6)
Al4—Ni1—Al4^ii^	146.06 (9)	Al3^ii^—Si1—Al4^ii^	132.04 (6)
Al5—Ni1—Al4^ii^	79.61 (7)	Al6^ii^—Si1—Al4^ii^	75.26 (6)
Si1^i^—Ni1—Al2^i^	64.40 (5)	Al6—Si1—Al4^ii^	112.98 (6)
Si1—Ni1—Al2^i^	117.29 (5)	Fe2—Si1—Al6^vii^	56.91 (4)
Al4—Ni1—Al2^i^	70.92 (4)	Ni2—Si1—Al6^vii^	56.91 (4)
Al5—Ni1—Al2^i^	105.37 (4)	Ni1—Si1—Al6^vii^	109.06 (5)
Al4^ii^—Ni1—Al2^i^	120.81 (4)	Fe1—Si1—Al6^vii^	109.06 (5)
Si1^i^—Ni1—Al2	117.29 (5)	Si1^i^—Si1—Al6^vii^	64.03 (3)
Si1—Ni1—Al2	64.40 (5)	Al2^ii^—Si1—Al6^vii^	119.82 (6)
Al4—Ni1—Al2	70.92 (4)	Al2—Si1—Al6^vii^	110.02 (6)
Al5—Ni1—Al2	105.37 (4)	Al3^ii^—Si1—Al6^vii^	73.70 (5)
Al4^ii^—Ni1—Al2	120.81 (4)	Al6^ii^—Si1—Al6^vii^	64.71 (7)
Al2^i^—Ni1—Al2	114.44 (7)	Al6—Si1—Al6^vii^	56.51 (7)
Si1^i^—Ni1—Al2^iii^	72.57 (4)	Al4^ii^—Si1—Al6^vii^	124.34 (5)
Si1—Ni1—Al2^iii^	126.33 (5)	Ni1—Al2—Ni1^iv^	115.73 (5)
Al4—Ni1—Al2^iii^	98.98 (4)	Ni1—Al2—Si1^xi^	102.07 (6)
Al5—Ni1—Al2^iii^	65.74 (4)	Ni1^iv^—Al2—Si1^xi^	133.31 (7)
Al4^ii^—Ni1—Al2^iii^	65.31 (4)	Fe1—Al2—Si1	55.02 (4)
Al2^i^—Ni1—Al2^iii^	64.27 (5)	Ni1—Al2—Si1	55.02 (4)
Al2—Ni1—Al2^iii^	169.06 (3)	Ni1^iv^—Al2—Si1	98.46 (6)
Si1^i^—Ni1—Al2^iv^	126.33 (5)	Si1^xi^—Al2—Si1	126.27 (7)
Si1—Ni1—Al2^iv^	72.57 (4)	Ni1—Al2—Al4^viii^	151.06 (7)
Al4—Ni1—Al2^iv^	98.98 (4)	Ni1^iv^—Al2—Al4^viii^	55.63 (5)
Al5—Ni1—Al2^iv^	65.74 (4)	Si1^xi^—Al2—Al4^viii^	77.96 (6)
Al4^ii^—Ni1—Al2^iv^	65.31 (4)	Si1—Al2—Al4^viii^	146.54 (7)
Al2^i^—Ni1—Al2^iv^	169.06 (3)	Ni1—Al2—Al5^iv^	130.35 (7)
Al2—Ni1—Al2^iv^	64.27 (5)	Ni1^iv^—Al2—Al5^iv^	55.04 (5)
Al2^iii^—Ni1—Al2^iv^	114.73 (7)	Si1^xi^—Al2—Al5^iv^	117.54 (7)
Si1^i^—Ni1—Al6	66.74 (5)	Si1—Al2—Al5^iv^	76.98 (6)
Si1—Ni1—Al6	66.74 (5)	Al4^viii^—Al2—Al5^iv^	70.69 (6)
Al4—Ni1—Al6	82.26 (6)	Ni1—Al2—Al2^iv^	58.09 (5)
Al5—Ni1—Al6	148.71 (7)	Ni1^iv^—Al2—Al2^iv^	57.64 (5)
Al4^ii^—Ni1—Al6	131.68 (7)	Si1^xi^—Al2—Al2^iv^	147.40 (9)
Al2^i^—Ni1—Al6	61.86 (4)	Si1—Al2—Al2^iv^	66.49 (6)
Al2—Ni1—Al6	61.86 (4)	Al4^viii^—Al2—Al2^iv^	106.78 (8)
Al2^iii^—Ni1—Al6	122.18 (3)	Al5^iv^—Al2—Al2^iv^	93.85 (8)
Al2^iv^—Ni1—Al6	122.18 (3)	Fe1—Al2—Al6	63.42 (5)
Si1^i^—Fe1—Si1	63.97 (7)	Ni1—Al2—Al6	63.42 (5)
Si1^i^—Fe1—Al4	133.68 (5)	Ni1^iv^—Al2—Al6	160.41 (7)
Si1—Fe1—Al4	133.68 (5)	Si1^xi^—Al2—Al6	62.04 (6)
Si1^i^—Fe1—Al5	136.66 (5)	Si1—Al2—Al6	64.36 (6)
Si1—Fe1—Al5	136.66 (5)	Al4^viii^—Al2—Al6	135.03 (8)
Al4—Fe1—Al5	66.45 (7)	Al5^iv^—Al2—Al6	109.22 (6)
Si1^i^—Fe1—Al4^ii^	72.66 (5)	Al2^iv^—Al2—Al6	117.91 (8)
Si1—Fe1—Al4^ii^	72.66 (5)	Fe1—Al2—Fe2	97.79 (5)
Al4—Fe1—Al4^ii^	146.06 (9)	Si1^xi^—Al2—Fe2	92.76 (5)
Al5—Fe1—Al4^ii^	79.61 (7)	Si1—Al2—Fe2	52.11 (4)
Si1^i^—Fe1—Al2^i^	64.40 (5)	Al4^viii^—Al2—Fe2	111.13 (6)
Si1—Fe1—Al2^i^	117.29 (5)	Al5^iv^—Al2—Fe2	54.17 (4)
Al4—Fe1—Al2^i^	70.92 (4)	Al2^iv^—Al2—Fe2	114.12 (7)
Al5—Fe1—Al2^i^	105.37 (4)	Al6—Al2—Fe2	55.20 (4)
Al4^ii^—Fe1—Al2^i^	120.81 (4)	Ni1—Al2—Ni2	97.79 (5)
Si1^i^—Fe1—Al2	117.29 (5)	Ni1^iv^—Al2—Ni2	107.38 (5)
Si1—Fe1—Al2	64.40 (5)	Si1^xi^—Al2—Ni2	92.76 (5)
Al4—Fe1—Al2	70.92 (4)	Si1—Al2—Ni2	52.11 (4)
Al5—Fe1—Al2	105.37 (4)	Al4^viii^—Al2—Ni2	111.13 (6)
Al4^ii^—Fe1—Al2	120.81 (4)	Al5^iv^—Al2—Ni2	54.17 (4)
Al2^i^—Fe1—Al2	114.44 (7)	Al2^iv^—Al2—Ni2	114.12 (7)
Si1^i^—Fe1—Al2^iii^	72.57 (4)	Al6—Al2—Ni2	55.20 (4)
Si1—Fe1—Al2^iii^	126.33 (5)	Ni2^xi^—Al3—Ni2	142.04 (9)
Al4—Fe1—Al2^iii^	98.98 (4)	Ni2^xi^—Al3—Si1^xi^	55.07 (4)
Al5—Fe1—Al2^iii^	65.74 (4)	Ni2—Al3—Si1^xi^	98.96 (6)
Al4^ii^—Fe1—Al2^iii^	65.31 (4)	Ni2^xi^—Al3—Si1^v^	98.96 (6)
Al2^i^—Fe1—Al2^iii^	64.27 (5)	Ni2—Al3—Si1^v^	55.07 (4)
Al2—Fe1—Al2^iii^	169.06 (3)	Si1^xi^—Al3—Si1^v^	100.28 (8)
Si1^i^—Fe1—Al2^iv^	126.33 (5)	Ni2^xi^—Al3—Al3^ix^	108.98 (5)
Si1—Fe1—Al2^iv^	72.57 (4)	Ni2—Al3—Al3^ix^	108.98 (5)
Al4—Fe1—Al2^iv^	98.98 (4)	Si1^xi^—Al3—Al3^ix^	129.86 (4)
Al5—Fe1—Al2^iv^	65.74 (4)	Si1^v^—Al3—Al3^ix^	129.86 (4)
Al4^ii^—Fe1—Al2^iv^	65.31 (4)	Ni2^xi^—Al3—Al5^viii^	53.52 (3)
Al2^i^—Fe1—Al2^iv^	169.06 (3)	Ni2—Al3—Al5^viii^	152.38 (4)
Al2—Fe1—Al2^iv^	64.27 (5)	Si1^xi^—Al3—Al5^viii^	72.49 (4)
Al2^iii^—Fe1—Al2^iv^	114.73 (7)	Si1^v^—Al3—Al5^viii^	151.08 (5)
Si1^i^—Fe1—Al6	66.74 (5)	Al3^ix^—Al3—Al5^viii^	63.38 (4)
Si1—Fe1—Al6	66.74 (5)	Ni2^xi^—Al3—Al5^vi^	152.38 (4)
Al4—Fe1—Al6	82.26 (6)	Ni2—Al3—Al5^vi^	53.52 (3)
Al5—Fe1—Al6	148.71 (7)	Si1^xi^—Al3—Al5^vi^	151.08 (5)
Al4^ii^—Fe1—Al6	131.68 (7)	Si1^v^—Al3—Al5^vi^	72.49 (4)
Al2^i^—Fe1—Al6	61.86 (4)	Al3^ix^—Al3—Al5^vi^	63.38 (4)
Al2—Fe1—Al6	61.86 (4)	Al5^viii^—Al3—Al5^vi^	126.76 (8)
Al2^iii^—Fe1—Al6	122.18 (3)	Ni1—Al4—Ni1^xi^	146.06 (9)
Al2^iv^—Fe1—Al6	122.18 (3)	Fe1—Al4—Al5	56.78 (6)
Si1—Ni2—Si1^v^	136.03 (7)	Ni1—Al4—Al5	56.78 (6)
Si1—Ni2—Al3^ii^	66.00 (4)	Ni1^xi^—Al4—Al5	157.15 (9)
Si1^v^—Ni2—Al3^ii^	130.56 (3)	Ni1—Al4—Al2^viii^	127.33 (4)
Si1—Ni2—Al3	130.56 (3)	Ni1^xi^—Al4—Al2^viii^	59.06 (5)
Si1^v^—Ni2—Al3	66.00 (4)	Al5—Al4—Al2^viii^	110.41 (6)
Al3^ii^—Ni2—Al3	142.04 (9)	Ni1—Al4—Al2^xii^	127.33 (4)
Si1—Ni2—Al5^vi^	134.89 (6)	Ni1^xi^—Al4—Al2^xii^	59.06 (5)
Si1^v^—Ni2—Al5^vi^	86.35 (5)	Al5—Al4—Al2^xii^	110.41 (6)
Al3^ii^—Ni2—Al5^vi^	74.21 (6)	Al2^viii^—Al4—Al2^xii^	105.30 (8)
Al3—Ni2—Al5^vi^	73.40 (6)	Ni1—Al4—Si1^xi^	97.76 (7)
Si1—Ni2—Al5^iv^	86.35 (5)	Ni1^xi^—Al4—Si1^xi^	52.59 (5)
Si1^v^—Ni2—Al5^iv^	134.89 (6)	Al5—Al4—Si1^xi^	144.72 (6)
Al3^ii^—Ni2—Al5^iv^	73.40 (6)	Al2^viii^—Al4—Si1^xi^	63.26 (5)
Al3—Ni2—Al5^iv^	74.21 (6)	Al2^xii^—Al4—Si1^xi^	104.53 (7)
Al5^vi^—Ni2—Al5^iv^	61.93 (8)	Ni1—Al4—Si1^xiii^	97.76 (7)
Si1—Ni2—Al6	71.17 (6)	Ni1^xi^—Al4—Si1^xiii^	52.59 (5)
Si1^v^—Ni2—Al6	71.94 (6)	Al5—Al4—Si1^xiii^	144.72 (6)
Al3^ii^—Ni2—Al6	132.41 (6)	Al2^viii^—Al4—Si1^xiii^	104.53 (7)
Al3—Ni2—Al6	82.83 (6)	Al2^xii^—Al4—Si1^xiii^	63.26 (5)
Al5^vi^—Ni2—Al6	152.89 (7)	Si1^xi^—Al4—Si1^xiii^	52.31 (6)
Al5^iv^—Ni2—Al6	123.99 (5)	Ni1—Al4—Al2^i^	56.25 (4)
Si1—Ni2—Al6^vii^	71.94 (6)	Ni1^xi^—Al4—Al2^i^	104.41 (6)
Si1^v^—Ni2—Al6^vii^	71.17 (6)	Al5—Al4—Al2^i^	90.76 (6)
Al3^ii^—Ni2—Al6^vii^	82.83 (6)	Al2^viii^—Al4—Al2^i^	156.54 (8)
Al3—Ni2—Al6^vii^	132.41 (6)	Al2^xii^—Al4—Al2^i^	75.01 (5)
Al5^vi^—Ni2—Al6^vii^	123.99 (5)	Si1^xi^—Al4—Al2^i^	93.68 (7)
Al5^iv^—Ni2—Al6^vii^	152.89 (7)	Si1^xiii^—Al4—Al2^i^	53.99 (4)
Al6—Ni2—Al6^vii^	64.66 (7)	Fe1—Al4—Al2	56.25 (4)
Si1—Ni2—Al2	61.25 (4)	Ni1—Al4—Al2	56.25 (4)
Si1^v^—Ni2—Al2	118.46 (4)	Ni1^xi^—Al4—Al2	104.41 (6)
Al3^ii^—Ni2—Al2	110.73 (3)	Al5—Al4—Al2	90.76 (6)
Al3—Ni2—Al2	69.51 (3)	Al2^viii^—Al4—Al2	75.01 (5)
Al5^vi^—Ni2—Al2	118.68 (5)	Al2^xii^—Al4—Al2	156.54 (8)
Al5^iv^—Ni2—Al2	61.98 (5)	Si1^xi^—Al4—Al2	53.99 (4)
Al6—Ni2—Al2	62.19 (5)	Si1^xiii^—Al4—Al2	93.68 (7)
Al6^vii^—Ni2—Al2	117.15 (5)	Al2^i^—Al4—Al2	95.41 (8)
Si1—Ni2—Al2^v^	118.46 (4)	Ni1—Al5—Ni2^xiv^	120.69 (4)
Si1^v^—Ni2—Al2^v^	61.25 (4)	Ni1—Al5—Ni2^iv^	120.69 (4)
Al3^ii^—Ni2—Al2^v^	69.51 (3)	Ni2^xiv^—Al5—Ni2^iv^	118.07 (8)
Al3—Ni2—Al2^v^	110.73 (3)	Ni1—Al5—Al5^x^	172.71 (14)
Al5^vi^—Ni2—Al2^v^	61.98 (5)	Ni2^xiv^—Al5—Al5^x^	59.03 (4)
Al5^iv^—Ni2—Al2^v^	118.68 (5)	Ni2^iv^—Al5—Al5^x^	59.03 (4)
Al6—Ni2—Al2^v^	117.15 (5)	Fe1—Al5—Al4	56.76 (6)
Al6^vii^—Ni2—Al2^v^	62.19 (5)	Ni1—Al5—Al4	56.76 (6)
Al2—Ni2—Al2^v^	179.31 (6)	Ni2^xiv^—Al5—Al4	109.53 (6)
Si1—Fe2—Si1^v^	136.03 (7)	Ni2^iv^—Al5—Al4	109.53 (6)
Si1—Fe2—Al3^ii^	66.00 (4)	Al5^x^—Al5—Al4	130.53 (13)
Si1^v^—Fe2—Al3^ii^	130.56 (3)	Ni1—Al5—Al2^iv^	59.22 (4)
Si1—Fe2—Al3	130.56 (3)	Ni2^xiv^—Al5—Al2^iv^	156.95 (9)
Si1^v^—Fe2—Al3	66.00 (4)	Ni2^iv^—Al5—Al2^iv^	63.84 (3)
Al3^ii^—Fe2—Al3	142.04 (9)	Al5^x^—Al5—Al2^iv^	117.76 (7)
Si1—Fe2—Al5^vi^	134.89 (6)	Al4—Al5—Al2^iv^	89.70 (6)
Si1^v^—Fe2—Al5^vi^	86.35 (5)	Ni1—Al5—Al2^iii^	59.22 (4)
Al3^ii^—Fe2—Al5^vi^	74.21 (6)	Ni2^xiv^—Al5—Al2^iii^	63.84 (3)
Al3—Fe2—Al5^vi^	73.40 (6)	Ni2^iv^—Al5—Al2^iii^	156.95 (9)
Si1—Fe2—Al5^iv^	86.35 (5)	Al5^x^—Al5—Al2^iii^	117.76 (7)
Si1^v^—Fe2—Al5^iv^	134.89 (6)	Al4—Al5—Al2^iii^	89.70 (6)
Al3^ii^—Fe2—Al5^iv^	73.40 (6)	Al2^iv^—Al5—Al2^iii^	105.04 (8)
Al3—Fe2—Al5^iv^	74.21 (6)	Ni1—Al5—Al3^viii^	121.16 (7)
Al5^vi^—Fe2—Al5^iv^	61.93 (8)	Ni2^xiv^—Al5—Al3^viii^	99.66 (7)
Si1—Fe2—Al6	71.17 (6)	Ni2^iv^—Al5—Al3^viii^	53.08 (4)
Si1^v^—Fe2—Al6	71.94 (6)	Al5^x^—Al5—Al3^viii^	65.12 (7)
Al3^ii^—Fe2—Al6	132.41 (6)	Al4—Al5—Al3^viii^	70.97 (6)
Al3—Fe2—Al6	82.83 (6)	Al2^iv^—Al5—Al3^viii^	98.58 (5)
Al5^vi^—Fe2—Al6	152.89 (7)	Al2^iii^—Al5—Al3^viii^	149.36 (7)
Al5^iv^—Fe2—Al6	123.99 (5)	Ni1—Al5—Al3^xiv^	121.16 (7)
Si1—Fe2—Al6^vii^	71.94 (6)	Ni2^xiv^—Al5—Al3^xiv^	53.08 (4)
Si1^v^—Fe2—Al6^vii^	71.17 (6)	Ni2^iv^—Al5—Al3^xiv^	99.66 (7)
Al3^ii^—Fe2—Al6^vii^	82.83 (6)	Al5^x^—Al5—Al3^xiv^	65.12 (7)
Al3—Fe2—Al6^vii^	132.41 (6)	Al4—Al5—Al3^xiv^	70.97 (6)
Al5^vi^—Fe2—Al6^vii^	123.99 (5)	Al2^iv^—Al5—Al3^xiv^	149.36 (7)
Al5^iv^—Fe2—Al6^vii^	152.89 (7)	Al2^iii^—Al5—Al3^xiv^	98.58 (5)
Al6—Fe2—Al6^vii^	64.66 (7)	Al3^viii^—Al5—Al3^xiv^	53.24 (8)
Si1—Fe2—Al2	61.25 (4)	Ni2—Al6—Ni2^vii^	115.34 (7)
Si1^v^—Fe2—Al2	118.46 (4)	Fe2—Al6—Al6^vii^	57.67 (4)
Al3^ii^—Fe2—Al2	110.73 (3)	Ni2—Al6—Al6^vii^	57.67 (4)
Al3—Fe2—Al2	69.51 (3)	Ni2^vii^—Al6—Al6^vii^	57.67 (4)
Al5^vi^—Fe2—Al2	118.68 (5)	Ni2—Al6—Al2^i^	148.81 (9)
Al5^iv^—Fe2—Al2	61.98 (5)	Ni2^vii^—Al6—Al2^i^	62.61 (3)
Al6—Fe2—Al2	62.19 (5)	Al6^vii^—Al6—Al2^i^	111.70 (7)
Al6^vii^—Fe2—Al2	117.15 (5)	Fe2—Al6—Al2	62.61 (3)
Si1—Fe2—Al2^v^	118.46 (4)	Ni2—Al6—Al2	62.61 (3)
Si1^v^—Fe2—Al2^v^	61.25 (4)	Ni2^vii^—Al6—Al2	148.81 (9)
Al3^ii^—Fe2—Al2^v^	69.51 (3)	Al6^vii^—Al6—Al2	111.70 (7)
Al3—Fe2—Al2^v^	110.73 (3)	Al2^i^—Al6—Al2	102.23 (8)
Al5^vi^—Fe2—Al2^v^	61.98 (5)	Ni2—Al6—Si1^xi^	94.03 (3)
Al5^iv^—Fe2—Al2^v^	118.68 (5)	Ni2^vii^—Al6—Si1^xi^	147.60 (6)
Al6—Fe2—Al2^v^	117.15 (5)	Al6^vii^—Al6—Si1^xi^	148.77 (6)
Al6^vii^—Fe2—Al2^v^	62.19 (5)	Al2^i^—Al6—Si1^xi^	99.42 (7)
Al2—Fe2—Al2^v^	179.31 (6)	Al2—Al6—Si1^xi^	56.67 (5)
Ni2—Si1—Ni1	114.09 (5)	Ni2—Al6—Si1^xiii^	147.60 (6)
Fe2—Si1—Fe1	114.09 (5)	Ni2^vii^—Al6—Si1^xiii^	94.03 (3)
Ni2—Si1—Si1^i^	111.99 (4)	Al6^vii^—Al6—Si1^xiii^	148.77 (6)
Ni1—Si1—Si1^i^	58.02 (3)	Al2^i^—Al6—Si1^xiii^	56.67 (5)
Ni2—Si1—Al2^ii^	126.96 (6)	Al2—Al6—Si1^xiii^	99.42 (7)
Ni1—Si1—Al2^ii^	115.40 (6)	Si1^xi^—Al6—Si1^xiii^	54.52 (6)
Si1^i^—Si1—Al2^ii^	109.44 (4)	Ni2—Al6—Ni1	96.91 (5)
Fe2—Si1—Al2	66.64 (4)	Ni2^vii^—Al6—Ni1	96.91 (5)
Ni2—Si1—Al2	66.64 (4)	Al6^vii^—Al6—Ni1	103.00 (10)
Ni1—Si1—Al2	60.58 (4)	Al2^i^—Al6—Ni1	54.73 (4)
Fe1—Si1—Al2	60.58 (4)	Al2—Al6—Ni1	54.73 (4)
Si1^i^—Si1—Al2	109.32 (4)	Si1^xi^—Al6—Ni1	92.54 (6)
Al2^ii^—Si1—Al2	126.27 (7)	Si1^xiii^—Al6—Ni1	92.54 (6)
Ni2—Si1—Al3^ii^	58.93 (3)	Fe2—Al6—Fe1	96.91 (5)
Ni1—Si1—Al3^ii^	170.13 (6)	Al6^vii^—Al6—Fe1	103.00 (10)
Si1^i^—Si1—Al3^ii^	129.86 (4)	Al2^i^—Al6—Fe1	54.73 (4)
Al2^ii^—Si1—Al3^ii^	69.38 (4)	Al2—Al6—Fe1	54.73 (4)
Al2—Si1—Al3^ii^	109.56 (6)	Si1^xi^—Al6—Fe1	92.54 (6)
Ni2—Si1—Al6^ii^	112.75 (6)	Si1^xiii^—Al6—Fe1	92.54 (6)
Ni1—Si1—Al6^ii^	113.97 (6)	Fe2—Al6—Si1	51.49 (4)
Si1^i^—Si1—Al6^ii^	62.74 (3)	Ni2—Al6—Si1	51.49 (4)
Al2^ii^—Si1—Al6^ii^	61.28 (5)	Ni2^vii^—Al6—Si1	97.14 (6)
Al2—Si1—Al6^ii^	171.58 (6)	Al6^vii^—Al6—Si1	62.24 (7)
Al3^ii^—Si1—Al6^ii^	75.85 (5)	Al2^i^—Al6—Si1	97.32 (7)
Fe2—Si1—Al6	57.33 (4)	Al2—Al6—Si1	55.90 (5)
Ni2—Si1—Al6	57.33 (4)	Si1^xi^—Al6—Si1	112.47 (6)
Ni1—Si1—Al6	63.21 (5)	Si1^xiii^—Al6—Si1	141.91 (9)
Fe1—Si1—Al6	63.21 (5)	Ni1—Al6—Si1	50.05 (4)
Si1^i^—Si1—Al6	63.77 (3)	Fe1—Al6—Si1	50.05 (4)
Al2^ii^—Si1—Al6	172.99 (6)		

Symmetry codes: (i) *x*, −*y*+1, *z*; (ii) *x*, *y*, *z*+1; (iii) −*x*+3/2, *y*+1/2, −*z*+1; (iv) −*x*+3/2, −*y*+1/2, −*z*+1; (v) −*x*+1, *y*, −*z*+1; (vi) *x*−1/2, *y*−1/2, *z*; (vii) −*x*+1, −*y*+1, −*z*+1; (viii) −*x*+3/2, −*y*+1/2, −*z*; (ix) −*x*+1, −*y*, −*z*; (x) −*x*+2, −*y*+1, −*z*+1; (xi) *x*, *y*, *z*−1; (xii) −*x*+3/2, *y*+1/2, −*z*; (xiii) *x*, −*y*+1, *z*−1; (xiv) *x*+1/2, *y*+1/2, *z*.

## References

[bb1] Brandenburg, K. & Putz, H. (2017). *DIAMOND*. Crystal Impact GbR, Bonn, Germany.

[bb2] Bruker (2023). *APEX5 and *SAINT**. Bruker AXS Inc. Madison, Wisconsin, USA, 2008.

[bb3] Cai, Q., Fang, C., Lordan, E., Wang, Y., Chang, I. & Cantor, B. (2023). *Scr. Mater.* **237**, 115707.

[bb4] Grin, J., Burkhardt, U., Ellner, M. & Peters, K. (1994). *Z. Kristallogr.* **209**, 479–487.

[bb10] Krause, L., Herbst-Irmer, R., Sheldrick, G. M. & Stalke, D. (2015). *J. Appl. Cryst.* **48**, 3–10. 10.1107/S1600576714022985 PMC4453166 26089746

[bb5] Liu, C. & Fan, C. (2018). *IUCrData* **3**, x180363.

[bb6] Nejadsattari, F., Stadnik, Z. M., Przewoźnik, J. & Grushko, B. (2016). *J. Alloys Compd.* **662**, 612–620.

[bb7] Sheldrick, G. M. (2015*a*). *Acta Cryst.* A**71**, 3–8.

[bb8] Sheldrick, G. M. (2015*b*). *Acta Cryst.* C**71**, 3–8.

[bb9] Westrip, S. P. (2010). *J. Appl. Cryst.* **43**, 920–925.

